# Mechanisms Underlying Dopaminergic Regulation of Nicotine-Induced Kinetic Tremor

**DOI:** 10.3389/fphar.2022.938175

**Published:** 2022-06-16

**Authors:** Masaki Kato, Naofumi Kunisawa, Saki Shimizu, Higor A. Iha, Yukihiro Ohno

**Affiliations:** Department of Pharmacology, Faculty of Pharmacy, Osaka Medical and Pharmaceutical University, Osaka, Japan

**Keywords:** nicotine, tremor, dopamine receptors, D_3_ receptors, inferior olive, cerebellum

## Abstract

Nicotine induces kinetic tremor, which resembles pharmacological features of essential tremors, via activating the inferior olive (IO) neurons. Since nicotine is known to enhance dopamine release by stimulating α4β2 and/or α6 nACh receptors, we examined the effects of various dopamine receptor ligands on nicotine-induced tremor to clarify the role of the dopaminergic system in modulating nicotine tremor. A tremorgenic dose of nicotine increased the dopamine level in the pons and medulla oblongata (P/MO), and the levels of dopamine metabolites in the hippocampus, P/MO, and striatum. Treatment of animals with the D_1/5_ agonist SKF-38393 inhibited the induction of nicotine tremor, whereas the D_3_ agonist PD-128,907 facilitated nicotine-induced tremor. The D_2_ agonist sumanirole showed no effect. In addition, nicotine tremor was significantly enhanced by the D_1/5_ antagonist SCH-23390 and inhibited by the D_3_ antagonist U-99194. Neither the D_2_ (L-741,626) nor D_4_ (L-745,870) antagonist affected the generation of nicotine tremor. Furthermore, microinjection of U-99194 into the cerebellum significantly inhibited nicotine-induced tremor, whereas its injection into IO or the striatum did not affect tremor generation. Although intrastriatal injection of SCH-23390 showed no effects, its injection into IO tended to enhance nicotine-induced tremor. The present study suggests that dopamine D_3_ and D_1/5_ receptors regulate the induction of nicotine tremor in an opposite way, D_3_ receptors facilitately and D_1/5_ receptors inhibitorily. In addition, the cerebellar D_3_ receptors may play an important role in modulating the induction of nicotine tremor mediated by the olivo-cerebellar system.

## Introduction

Nicotine, an active ingredient of tobacco products, produces a variety of pharmacological effects, including addictive actions (Le Foll and Goldberg, 2009; Besson et al., 2012; Harrington et al., 2016), antidepressant effects (Vieyra-Reyes et al., 2008; Mineur and Picciotto, 2010; Haj-Mirzaian et al., 2015), cognitive enhancement (Swan and Lessov-Schlaggar, 2007; Wood et al., 2016), and motor disturbances (e.g., tremor and seizures) (Lippold et al., 1980; Shiffman et al., 1983; Miner et al., 1985; Louis, 2007; Lin et al., 2012; Gilley and Beno, 2020). These actions are generally considered to be mediated by nicotinic acetylcholine (nACh) receptors, particularly α7 and α4β2 receptors, which are major subtypes in the brain (Tuesta et al., 2011; Besson et al., 2012; Koukouli and Maskos, 2015; Wood et al., 2016). In addition, it has been shown that nicotine facilitates synaptic dopamine release via activating α4β2 receptors and/or nACh receptors containing α6 subunits, which are abundantly expressed in the mesolimbic and nigrostriatal dopaminergic neurons (Damsma et al., 1988; Haikala and Ahtee, 1988; Wonnacott, 1997; Wonnacott et al., 2000; Exley et al., 2008; Quik et al., 2011). The activation of dopaminergic neurotransmission by nicotine is closely associated with its psychoemotional actions (e.g., addictive and antidepressant effects); however, the role of the dopaminergic system in modulating nicotine-induced motor disturbances (e.g., tremor) remains unknown.

Tremor is an involuntary and rhythmic movement disorder which appears in different parts of the body. According to the clinical manifestation, tremor can be classified into several types: 1) resting tremor occurring in the resting state of the limb, 2) kinetic tremor which appears during movement of parts of the body, 3) postural tremor which occurs when the body part is voluntarily maintained against gravity (holding a specific posture) (Puschmann and Wszolek, 2011; Kosmowska and Wardas, 2021). Resting tremor is one of the typical symptoms of Parkinson’s disease, while kinetic and postural tremors are frequently manifested in patients with essential tremor. In addition, several drugs, discretionary products (e.g., coffee and cigarettes), and toxins are known to induce tremor.

Previous studies reported that cigarette smoking and oral nicotine induce hand tremor (Lippold et al., 1980; Shiffman et al., 1983; Louis, 2007; Lin et al., 2012) and worsen essential tremor (Marshall and Schnieden, 1966). We previously showed that nicotine at relatively low doses (0.5–1 mg/kg, i.p.) evokes kinetic tremor in rodents (Kunisawa et al., 2016; Kunisawa et al., 2017; Kunisawa et al., 2018). Immunohistochemical analysis of Fos protein expression, a biological marker of neural excitation (Iha et al., 2016), revealed that tremorgenic doses of nicotine region-specifically increased the activities of the inferior olive (IO) neurons (Kunisawa et al., 2016) similarly to the rat model of essential tremor (Ohno et al., 2015a). In addition, electrical lesion of IO suppressed the induction of nicotine tremor, illustrating that nicotine induces kinetic tremor by activating IO neurons. Since excitation of the olivo-cerebellar neural pathway is reportedly involved in generation of essential tremor in humans (Puschmann and Wszolek, 2011; Kosmowska and Wardas, 2021; Welton et al., 2021), our findings suggest that nicotine-induced tremor has the same neural basis as essential tremor. In fact, nicotine tremor was significantly alleviated by drugs effective for human essential tremor (e.g., propranolol, diazepam, and phenobarbital), but was unaffected by medications for Parkinson’s disease tremor (i.e., trihexyphenidyl) (Kunisawa et al., 2018). Thus, nicotine-induced tremor may serve as an animal model of essential tremor. Interestingly, we found several factors which can modulate the intensity of nicotine tremor, including 5-HT_1A_ and 5-HT_2_ receptors, adrenergic β receptors, Na_v1_ channels, Ca_v3_ channels, and GABA_A_ receptors. (Ohno et al., 2015a; Kunisawa et al., 2017; Kunisawa et al., 2018).

In the present study, we examined the effects of various dopamine receptor ligands on nicotine-induced tremor to explore the role and mechanism of the dopaminergic system in the generation of nicotine tremor. Since blockade of D_3_ receptors or stimulation of D_1/5_ receptors was found to reduce nicotine-induced tremor, D_3_ and D_1/5_ receptors may be involved in regulation of the induction of kinetic tremor.

## Materials and Methods

### Animals

Male ddY mice and SD rats (Japan SLC, Shizuoka, Japan) at 7–11 weeks of age were used. The animals were given food and water *ad libitum* and kept in air-conditioned rooms (24 ± 2°C and 55 ± 10% relative humidity) under a 12 h light/dark cycle (light on at 8:00 a.m.). The animal care and housing methods complied with the Guide for the Care and Use of Laboratory Animals of the Ministry of Education, Science, Sports and Culture of Japan. The experimental protocols were approved by the Experimental Animal Research Committee at Osaka Medical and Pharmaceutical University.

### Analysis of Brain Dopamine and its Metabolite Levels

A tremorgenic dose (1 mg/kg, i.p.) of nicotine was injected into mice and, 15 min later, brains were removed from the skull under anesthesia (pentobarbital: 80 mg/kg, i.p.). Each brain was immediately washed with ice-cold saline and dissected into 8 regions (cerebral cortex, hippocampus, striatum, thalamus, hypothalamus, midbrain, pons and medulla oblongata (P/MO), and cerebellum) on an ice-cold petri dish. Tissue samples were then weighed, suspended in a 20% (w/v) volume of 0.1 M perchloric acid solution containing 1 ng/μl isoproterenol (internal standard), and homogenized (1,500 rpm × 10 strokes). After centrifugation (20,000 g × 15 min) at 0°C, the supernatant was filtered, and 35 μl of 1 M sodium acetate solution was added to 200 μl of the filtrate. Dopamine and its metabolites, 3,4-Dihydroxyphenylacetic acid (DOPAC) and homovanillic acid (HVA), in the extracts were measured using the HPLC-ECD system (Eicom, Kyoto, Japan). Namely, 1 μl of each sample were diluted with 99 μl 0.02 M acetic acid, and 10 μl of diluted samples were injected by an auto injector (M-500, Eicom, Kyoto, Japan). Dopamine, DOPAC, and HVA were separated by 3.0φ × 150 mm reversed-phase column (EICOMPAK SC-5ODS, Eicom). The column temperature was constantly held at 25°C with a column oven (ATC-300, Eicom). The mobile phase consisted of 0.1 M acetate-citrate buffer, 190 mg/L sodium 1-octanesulfonate, 5 mg/L EDTA, pH 3.5, and 17% methanol pumped at a flow rate of 500 μl/min by a reciprocal dual piston pump (EP-700, Eicom). The detector potential was +750 mV vs. Ag/AgCl (ECD-700A, Eicom). All data were analyzed by using a data processor (EPC-500, Eicom) and PowerChrom software (eDAQ Pty Ltd, Denistone East, NSW, Australia). Dopamine, DOPAC, and HVA were identified based on their retention times.

### Evaluation of Nicotine-Induced Tremor in Mice

Nicotine-induced tremor was evaluated as reported previously (Kunisawa et al., 2018). Briefly, mice were treated with a tremorgenic dose (1 mg/kg, i.p.) of nicotine and individually placed in an observation cage (16 × 24 × 12 cm). The intensity and duration of nicotine-induced tremor were measured in a time-sampling manner over 1-2, 3-4, 5-6, 7-8, and 9–10 min (each for 1-min measurement) after the nicotine injection. The tremor intensity was measured using 4-point ranked scoring (0: no tremor, 1: mild tremor in head and tail, and straub tail, 2: moderate tremor in upper trunk, 3: marked tremor in whole body). The total tremor score and tremor duration were calculated as a sum of the tremor score or duration time per point, respectively.

To evaluate the effects of dopamine receptor agonists on nicotine tremor, the following agents were administered intraperitoneally 15 min before the nicotine injection: the D_1/5_ receptor agonist SKF-38393 (3, 10 mg/kg), selective D_2_ receptor agonist sumanirole (1, 3 mg/kg), or selective D_3_ receptor agonist PD-128,907 (1, 3 mg/kg). The D_1/5_ receptor antagonist SCH-23390 (1, 3 mg/kg), selective D_2_ receptor antagonist L-741,626 (3, 10 mg/kg), selective D_3_ receptor antagonist U-99194 (30 mg/kg), or selective D_4_ receptor antagonist L-745,870 (0.3, 1 mg/kg) were also administered intraperitoneally as dopamine antagonists 15 min before the nicotine treatment. The dosage of each dopamine agonist or antagonist was set to effectively act on each dopamine receptor according to previous reports (SKF-38393 and SCH-23390; Ohno et al., 1997, sumanirole; Crans et al., 2020; PD-128,907; Boulay et al., 1999; L-741,626; Manvich et al., 2019; U-99194; Barth et al., 2013, L-745,870; Bernaerts and Tirelli, 2003).

### Microinjection Experiments With Dopamine Antagonists in Rats

Microinjection experiments were performed in rats as reported previously (Shimizu et al., 2013; 2014). Briefly, rats were anesthetized with pentobarbital (45 mg/kg, i.p.) and isoflurane inhalation, and the brain was fixed to a stereotaxic apparatus (Narishige, Tokyo, Japan). For the intraolivar or intrastriatal microinjection, two small holes were made in the skull, and bilateral stainless guide cannulae were implanted 1 mm above IO (AP: –12.4 mm, LM: ±0.5 mm, DV: +6.8 mm) or the striatum (AP: +1.0 mm, LM: ±3.5 mm, DV: +2.5 mm), respectively (Paxinos and Watson, 2007). Thereafter, guide cannulae were fixed to the skull with dental cement. For the intracerebellar microinjection, one guide cannula was implanted 1 mm above lobe IX of the cerebellum (AP: –13.1 mm, LM: ±0.0 mm, DV: +3.0 mm) (Paxinos and Watson, 2007).

After a recovery period of about 1 week, microinjection experiments with dopamine antagonists were performed. Under freely moving conditions, injection cannulae filled with SCH-23390 (3 μg/μl), U-99194 (30 μg/μl), or saline solution (control) were inserted into IO, the striatum, or cerebellum via guide cannulae, and 1 μl/site of the drug solution was gradually injected into the target site at a flow rate of 0.25 μl/min for 4 min using a microinfusion pump (KDS220, KD scientific, Holliston, MA, United States). Fifteen minutes after finishing the microinjection, the rats were treated with nicotine (1 mg/kg, i.p.) and behavioral observations of nicotine-induced tremor were performed during 5–16 min after the nicotine treatment, as described previously. The microinjection dosage of each dopamine antagonist was set to effectively act on each dopamine receptor according to previous reports (SCH-23390; Pardey et al., 2013, U-99194; Shimizu et al., 2014).

### Drugs

(–)-Nicotine and R(+)-SCH-23390 hydrochloride were purchased from Sigma-Aldrich (St. Louis, MO, United States). L-741,626, L-745,870 trihydrochloride, and (±)-SKF-38393 hydrochloride were purchased from Abcam (Cambridge, United Kingdom). U-99194 maleate, sumanirole maleate, and (+)-PD-128,907 hydrochloride were purchased from Tocris Bioscience (Bristol, United Kingdom). L-741,626 and L-745,870 were first dissolved in 1% lactate, and then they were diluted with distilled water. Other drugs were dissolved in saline.

### Statistical Analyses

Data are presented as the mean ± S.E.M. Significant differences on two-group comparisons were determined by Student’s *t*-test (parametric analysis) or the Mann-Whitney’s *U*-test (non-parametric analysis). For multigroup comparisons, the Kruskal-Wallis test followed by Steel-Dwass post-hoc multiple comparison test was performed. When the *p*-value was less than 0.05, it was considered significant.

## Results

### Effects of Tremorgenic Nicotine on Brain Dopamine and its Metabolite Levels

To investigate the changes in brain dopamine and its metabolite levels by nicotine, we treated mice with a tremorgenic dose (1 mg/kg, i.p.) of nicotine and measured the levels of dopamine, DOPAC, and HVA in 8 brain regions.

Nicotine significantly increased the dopamine level in P/MO as compared with control animals (*t* = 5.1374, *df* = 12, *p* = 0.0002) (Table 1). The dopamine level also tended to increase with nicotine in the striatum and hippocampus (Table 1). The level of DOPAC was significantly elevated in the hippocampus (*t* = 3.9544, *df* = 12, *p* = 0.0019) and P/MO (*t* = 2.3037, *df* = 12, *p* = 0.0399) (Table 1). Moreover, the HVA level was significantly increased in the striatum (*t* = 3.7921, *df* = 12, *p* = 0.0026) (Table 1).

**TABLE 1:** Changes in brain dopamine and its metabolite levels by a tremorgenic dose of nicotine in mice.

Brain regions	—	Level (ng/g tissue)		
		DA	DOPAC	HVA
Cerebral cortex	Saline	1,150.2 ± 198.2	144.4 ± 18.5	186.1 ± 23.8
	Nicotine	824.6 ± 83.6	151.2 ± 8.0	166.7 ± 14.6
Hippocampus	Saline	18.6 ± 3.2	9.4 ± 1.1	9.2 ± 1.8
	Nicotine	27.9 ± 7.8	18.1 ± 1.9**	12.5 ± 1.6
Striatum	Saline	7,467.0 ± 656.4	649.4 ± 54.5	745.9 ± 59.9
	Nicotine	8,404.6 ± 505.2	769.1 ± 58.8	1,137.8 ± 84.2**
Thalamus	Saline	58.8 ± 14.1	59.7 ± 8.9	129.2 ± 9.6
	Nicotine	91.8 ± 19.4	90.8 ± 11.6	134.7 ± 11.7
Hypothalamus	Saline	336.6 ± 45.6	159.9 ± 22.4	156.7 ± 9.5
	Nicotine	377.7 ± 25.2	193.7 ± 15.9	149.1 ± 7.9
Midbrain	Saline	112.6 ± 32.2	64.6 ± 7.5	94.3 ± 8.2
	Nicotine	114.0 ± 13.8	80.0 ± 8.9	87.4 ± 6.8
Pons and medulla oblongata	Saline	19.8 ± 1.6	34.0 ± 4.0	9.3 ± 1.9
	Nicotine	30.7 ± 1.4**	47.6 ± 4.3*	9.6 ± 1.7
Cerebellum	Saline	5.8 ± 0.3	7.7 ± 1.0	2.5 ± 0.2
	Nicotine	5.3 ± 0.3	7.8 ± 0.6	2.8 ± 0.3

Brain samples were obtained 15 min after the injection of nicotine (1 mg/kg, i.p.) or saline. Dopamine (DA), DOPAC, and HVA, levels were measured by the HPLC-ECD, system. Each value represents the mean ± S.E.M. *p < 0.05, **p < 0.01: Significantly different from control (saline treated) animals.

### Effects of Dopamine Receptor Agonists on Nicotine-Induced Tremor

**FIGURE 1:**
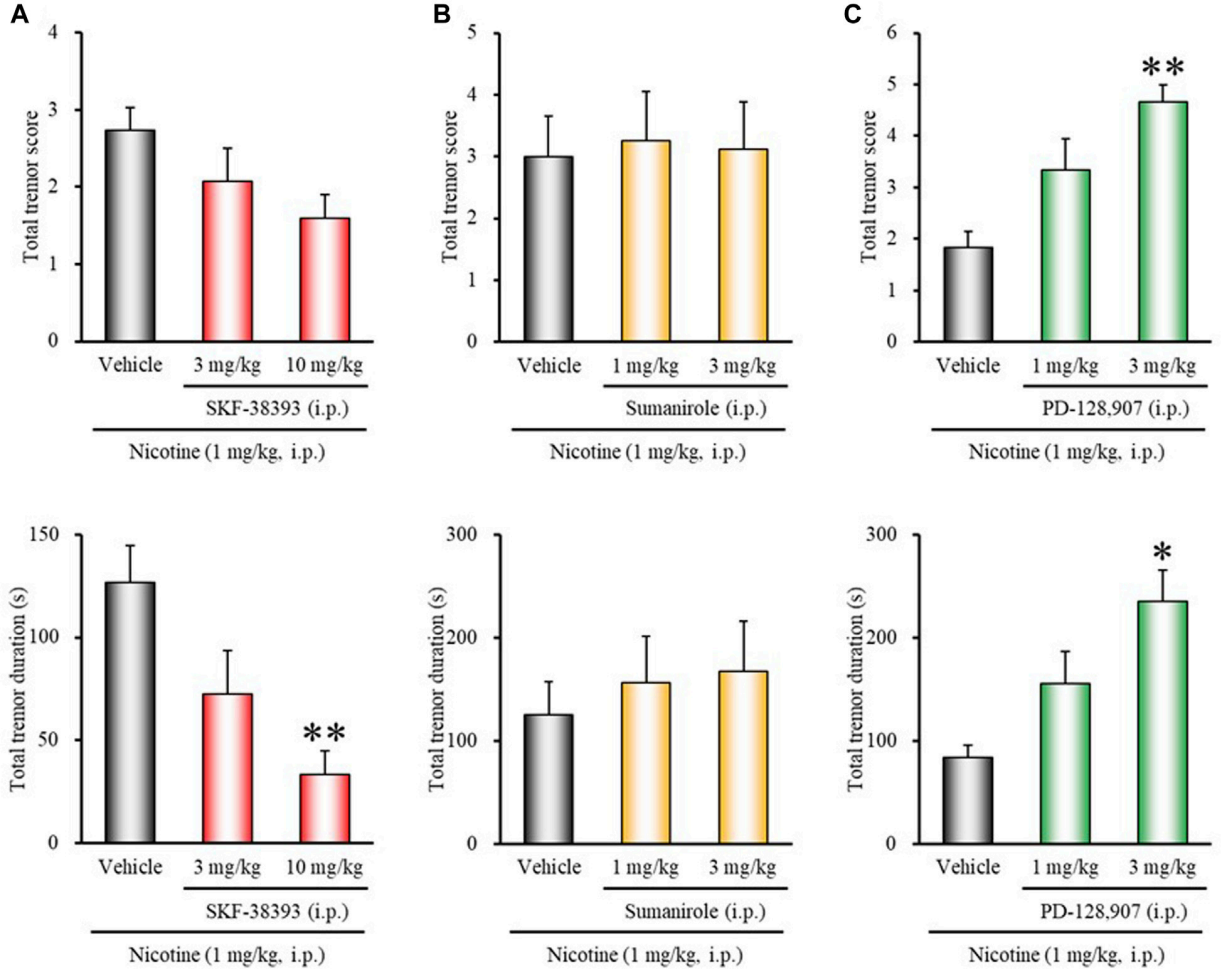
Effects of dopamine receptor agonists on nicotine-induced tremor in mice. Mice were treated with: **(A)** the D_1/5_ agonist SKF-38393 (3, 10 mg/kg, i.p.), **(B)** the D_2_ agonist sumanirole (1, 3 mg/kg, i.p.), **(C)** the D_3_ agonist PD-128,907 (1, 3 mg/kg, i.p.), or vehicle (control) 15 min before the nicotine (1 mg/kg, i.p.) injection. Data show the total tremor score (intensity) and duration of nicotine-induced tremor over the 10 min observation period. Each column represents the mean ± S.E.M. *p < 0.05, **p < 0.01: Significantly different from control animals.

To clarify the role of dopamine receptor subtypes in modulating nicotine tremor, we first examined the effects of various dopamine receptor agonists on nicotine-induced tremor. As shown in Figure 1, treatment of mice with the D_1/5_ receptor agonist SKF-38393 (3, 10 mg/kg, i.p.) inhibited nicotine (1 mg/kg, i.p.)-induced tremor in a dose-dependent manner. The total tremor duration was markedly reduced by 10 mg/kg of SKF-38393 (χ^2^ = 13.9752, *df* = 2, *p* = 0.0009). In contrast, the selective D_3_ receptor agonist PD-128,907 significantly increased the total tremor score and duration of nicotine tremor (total tremor score: χ^2^ = 10.0783, *df* = 2, *p* = 0.0065; total tremor duration: χ^2^ = 8.8997, *df* = 2, *p* = 0.0117). The D_2_ selective agonist sumanirole did not affect the induction of nicotine tremor (Figure 1B).

### Effects of Dopamine Receptor Antagonists on Nicotine-Induced Tremor

**FIGURE 2:**
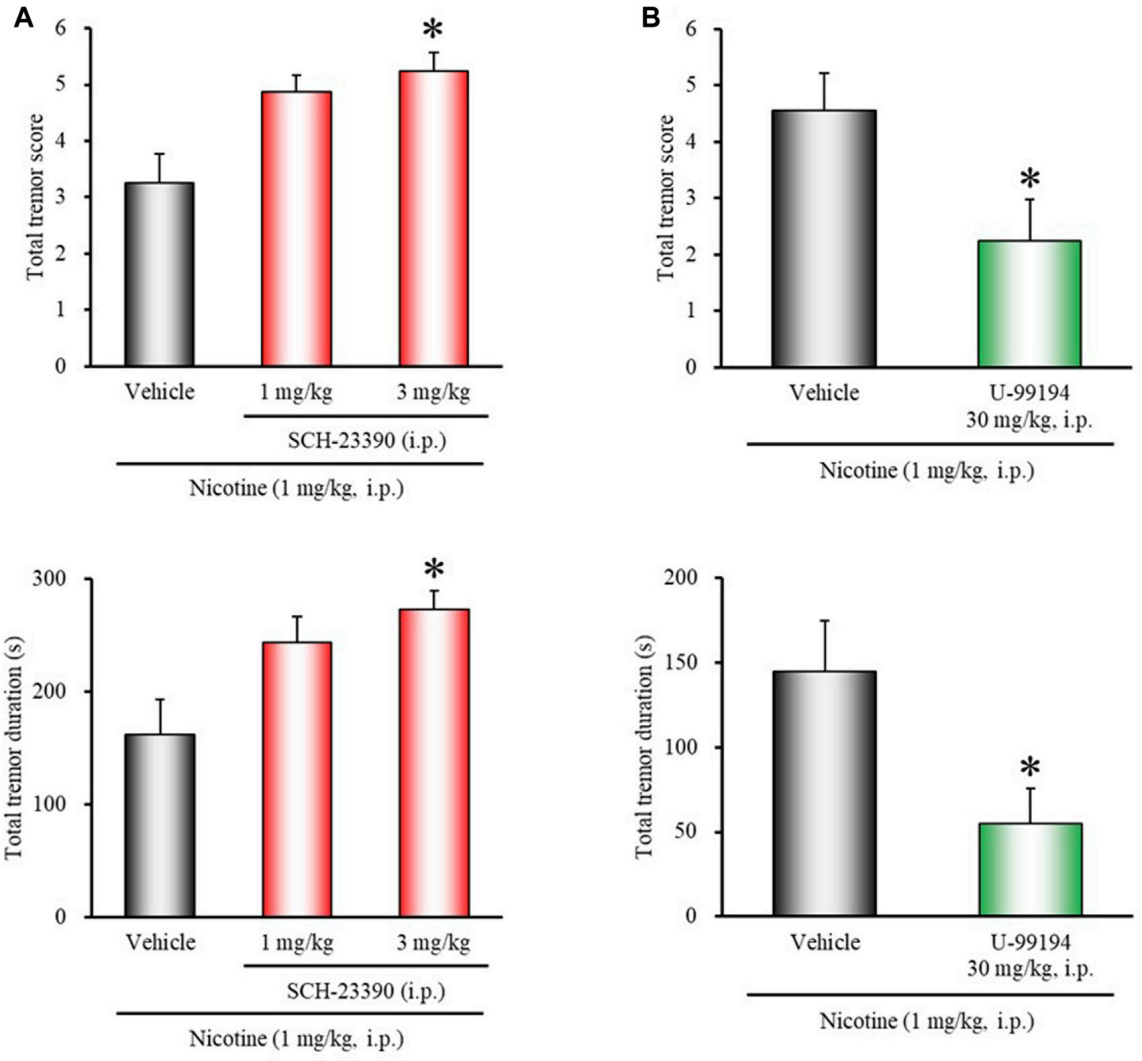
Effects of D_1/5_ or D_3_ receptor antagonists on nicotine-induced tremor in mice. Mice were treated with: **(A)** the D_1/5_ antagonist SCH-23390 (1, 3 mg/kg, i.p.), **(B)** the D_3_ antagonist U-99194 (30 mg/kg, i.p.), or vehicle (control) 15 min before the nicotine (1 mg/kg, i.p.) injection. Data show the total tremor score (intensity) and duration of nicotine-induced tremor over the 10 min observation period. Each column represents the mean ± S.E.M. *p < 0.05: Significantly different from control animals.

**FIGURE 3:**
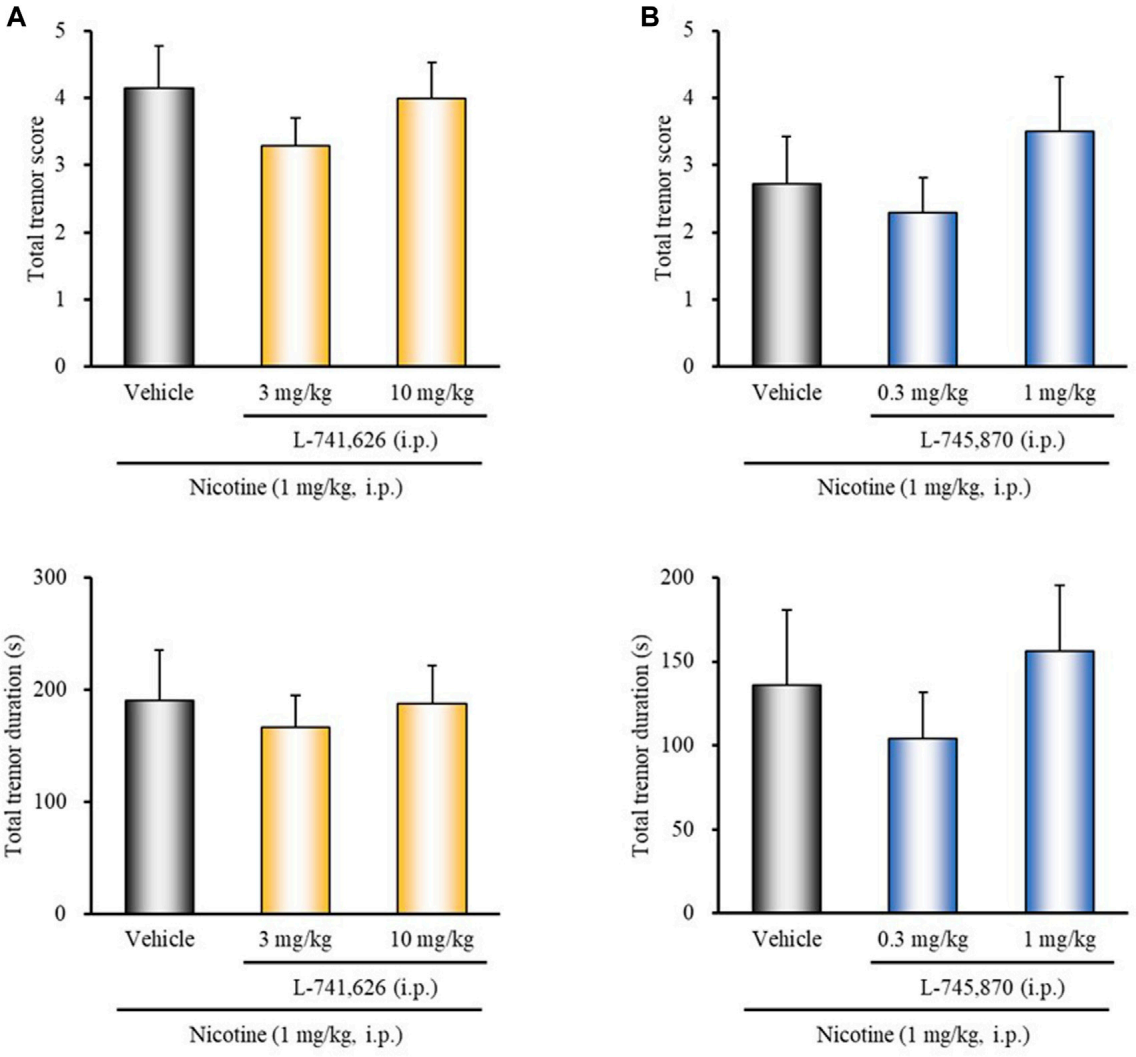
Effects of D_2_ or D_4_ receptor antagonists on nicotine-induced tremor in mice. Mice were treated with: **(A)** the D_2_ antagonist L-741,626 (3, 10 mg/kg, i.p.), **(B)** the D_4_ antagonist L-745,870 (0.3, 1 mg/kg, i.p.), or vehicle (control) 15 min before the nicotine (1 mg/kg, i.p.) injection. Data show the total tremor score (intensity) and duration of nicotine-induced tremor over the 10 min observation period. Each column represents the mean ± S.E.M.

To determine whether endogenous dopamine is involved in modulating nicotine tremor, we examined the direct effects of dopamine receptor antagonists on nicotine-induced tremor. As shown in Figure 2A, treatment of mice with the D_1/5_ antagonist SCH-23390 (3 mg/kg, i.p.) significantly potentiated nicotine-induced tremor (total tremor score: χ^2^ = 8.4150, *df* = 2, *p* = 0.0149; total tremor duration: χ^2^ = 8.5796, *df* = 2, *p* = 0.0137). Conversely, the D_3_ selective antagonist U-99194 (30 mg/kg, i.p.) significantly suppressed the nicotine tremor (total tremor score: *z* = 1.9646, *p* = 0.0495; total tremor duration: *z* = 2.1704, *p* = 0.0300) (Figure 2B). Nicotine-induced tremor, however, was unaffected either with the D_2_ selective antagonist L-741,626 or the D_4_ selective antagonist L-745,870 (Figure 3).

### Microinjection Studies With Dopamine Receptor Antagonists

To explore the regions of D_1/5_ and D_3_ receptors for regulation of nicotine-induced tremor, we conducted microinjection experiments using D_1/5_ (SCH-23390) and D_3_ (U-99194) antagonists in rats. Since IO and the striatum are important sites for the induction of nicotine tremor (Kunisawa et al., 2016) and parkinsonian tremor (Ohno et al., 2013; 2015b), respectively, SCH-23390 or U-99194 was injected into these two regions. In addition, as D_3_ receptors are highly expressed in the cerebellum and known to regulate locomotion and extrapyramidal movement disorders (Barik and de Beaurepaire, 1996, 2005; Kolasiewicz et al., 2008; Shimizu et al., 2014), U-99194 was also injected into the cerebellum (lobe IX).

**FIGURE 4:**
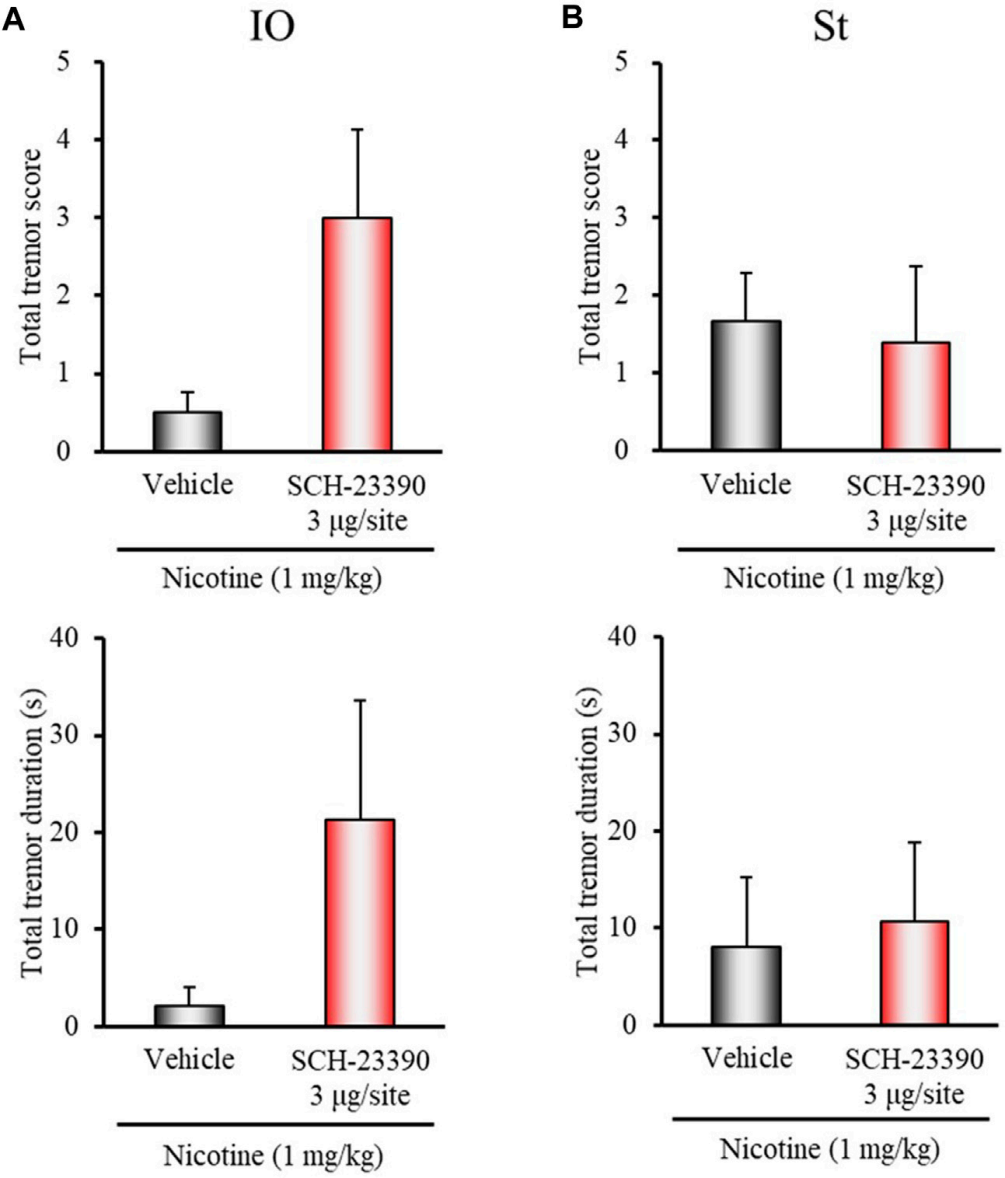
Effects of brain microinjection of SCH-23390 on nicotine-induced tremor in rats. The D_1/5_ antagonist SCH-23390 (3 μg/injection site) or vehicle (control) was injected into: **(A)** IO or **(B)** the striatum (St) in rats 15 min before the nicotine (1 mg/kg, i.p.) injection. Data show the total tremor score (intensity) and duration of nico tine-induced tremor during the 5–16 min observation period. Each column represents the mean ± S.E.M.

**FIGURE 5:**
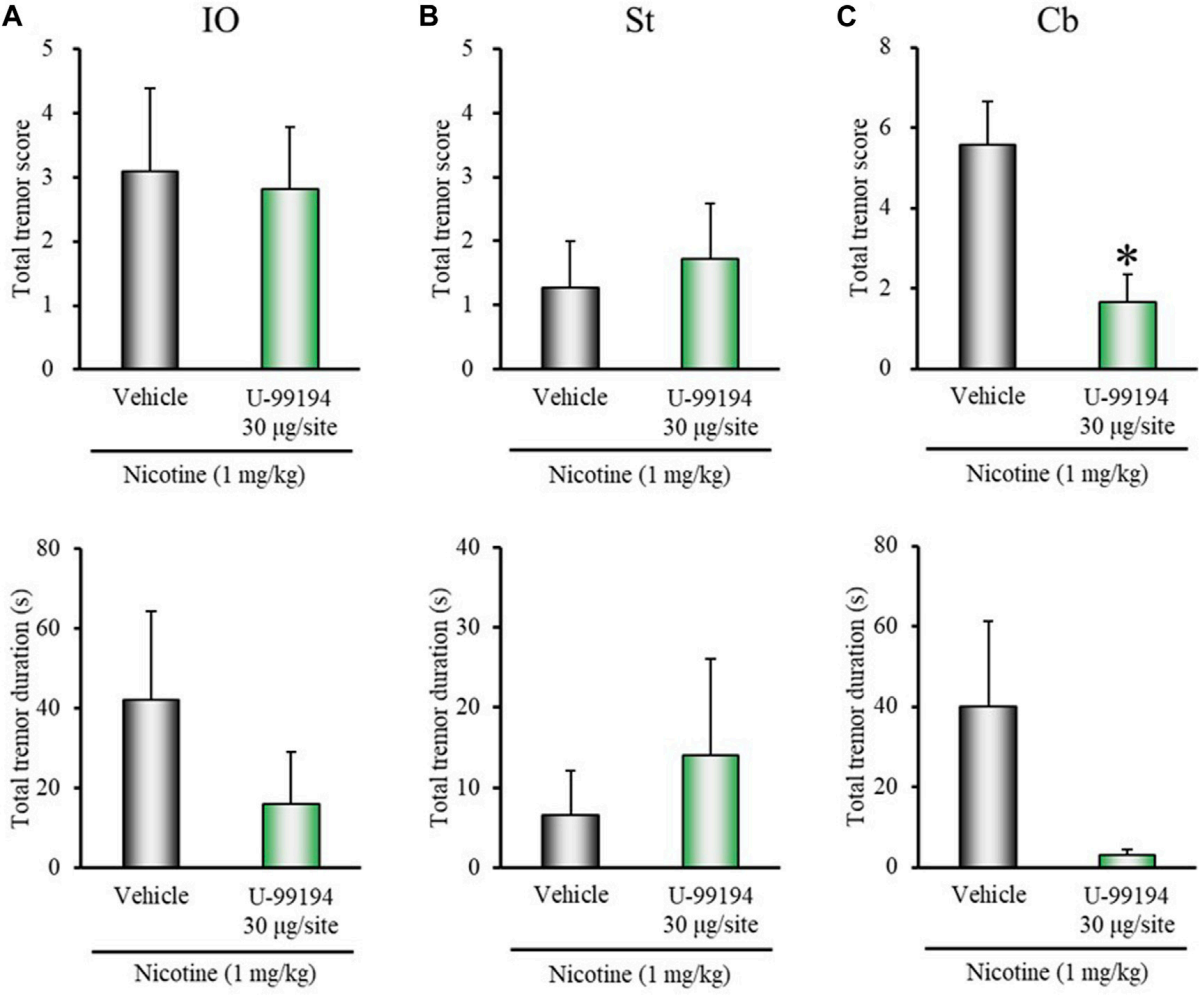
Effects of brain microinjection of U-99194 on nicotine-induced tremor in rats. The D_3_ antagonist U-99194 (30 μg/injection site) or vehicle (control) was injected into: **(A)** IO, **(B)** the striatum (St), or **(C)** the cerebellum lobe IX (Cb) in rats 15 min before the nicotine (1 mg/kg, i.p.) injection. Data show the total tremor score (intensity) and duration of nicotine-induced tremor during the 5–16 min observation period. Each column represents the mean ± S.E.M. *p < 0.05: Significantly different from control animals.

Microinjection of SCH-23390 (3 μg/injection site) into IO tended to enhance nicotine-induced tremor, although the changes did not reach significance (total tremor score: *z* = 1.7400, *p* = 0.0819; total tremor duration: *z* = 1.6963, *p* = 0.0898) (Figure 4A). Microinjection of SCH-23390 (3 μg/injection site) into the striatum did not affect the induction of nicotine tremor (Figure 4B). Furthermore, although microinjection of U-99194 into IO or the striatum failed to affect nicotine-induced tremor, its injection into the cerebellum markedly inhibited the induction of nicotine tremor (total tremor score: *z* = 2.4488, *p* = 0.0143; total tremor duration: *z* = 1.9393, *p* = 0.0525) (Figure 5).

## Discussion

Nicotine increases dopamine release and enhances dopaminergic neurotransmission in several brain regions (e.g., striatum, nucleus accumbens, and ventral tegmental area), which are suggested to be mediated by α4β2, α6β2, and partly α7 nACh receptors (Damsma et al., 1988; Haikala and Ahtee, 1988; Wonnacott, 1997; Wonnacott et al., 2000; Exley et al., 2008; Quik et al., 2011; Besson et al., 2012; Harrington et al., 2016). Previous studies showed that nicotine weakly increase the level of dopamine metabolites (e.g., DOPAC) by about 20–30% without affecting DA levels (Tani et al., 1997; Suemaru et al., 2006). The present study confirmed that nicotine at a tremorgenic dose increased dopamine and its metabolite levels in several parts of the brain (e.g., striatum, hippocampus, and P/MO), suggesting activation of the dopaminergic system by nicotine in these regions. Our results of nicotine actions on the striatal and hippocampal dopamine metabolism are similar to those in above previous studies. Meanwhile, in P/MO, nicotine elevated both dopamine and DOPAC levels by about 40–50%. Although the reason why nicotine elevated the DA level in P/MO remains uncertain, the effects of nicotine on dopamine neurons may involve its interactions with the serotonergic and/or noradrenergic systems (Gros et al., 2021; Pierucci et al., 2022). Although we did not specifically measure the dopamine level in IO, it is of interest that nicotine increased the dopaminergic tone in the P/MO region containing IO that forms the olivo-cerebellar neural circuit regulating tremor induction (Puschmann and Wszolek, 2011; Ohno et al., 2015a; Kosmowska and Wardas, 2021).

The present study using selective dopamine receptor ligands revealed that activation of D_3_ receptors facilitates the induction of nicotine tremor, whereas D_1/5_ receptor stimulation negatively regulates nicotine-induced tremor. Although D_3_ receptors belong to the D_2_ receptor family (Prieto, 2017; Kosmowska and Wardas, 2021), selective stimulation of D_2_ receptors by sumanirole showed no effects. Furthermore, since D_1/5_ and D_3_ antagonists per se enhanced and inhibited nicotine-induced tremor, respectively, activation of these receptors by endogenous dopamine may be involved in the regulation of nicotine tremor induction. It was confirmed that selective block of D_2_ or D_4_ receptors did not affect the induction of nicotine tremor, indicating that only D_3_ receptors in the D_2_ receptor family actively modulate the induction of kinetic tremor. Although we cannot completely deny the possibility that multiple actions of dopamine ligands used (e.g., interaction of SCH-23390 with 5-HT_2_ receptors and partial antagonism of SKF-38393 at D_1/5_ receptors) (Hoyer et al., 1989; Desai et al., 2005) also affected nicotine-induced tremor, our results suggest that dopamine D_3_ and D_1/5_ receptors regulate the induction of nicotine tremor in an opposite way, D_3_ receptors facilitately and D_1/5_ receptors inhibitorily.

We previously demonstrated that electrical lesion of IO suppressed nicotine-induced tremor (Kunisawa et al., 2016). Consistent with the fact that IO is closely involved in the pathogenesis of essential tremor in humans (Puschmann and Wszolek, 2011; Kosmowska and Wardas, 2021; Welton et al., 2021), our results suggest that the IO-cerebellar system plays a crucial role in the generation of nicotine-induced kinetic tremor. IO receives dopaminergic innervation from the ventral tegmental and prerubral parafascicular areas (Fallon et al., 1984; Toonen et al., 1998; Kitahama et al., 2000), suggesting a potential role of dopaminergic neurons in modulating tremor induction. Although microinjection of D_1/5_ and D_3_ antagonists into IO did not significantly affect nicotine tremor, the D_1/5_ antagonist markedly increased the intensity and duration of nicotine tremor. Our results suggest that D_1/5_ receptors in IO may be partly involved in modulation of nicotine tremor induction. Other D_1/5_ receptor regions involved in regulation of nicotine tremor remain to be clarified.

It is well-known that striatal dopamine receptors, particularly D_2_ receptors, play a crucial role in regulation of extrapyramidal motor functions and induction of parkinsonian symptoms, including resting tremor (Ohno et al., 2013; Ohno et al., 2015b). In the present study, however, the selective D_2_ receptor agonist sumanirole did not affect the generation of nicotine-induced kinetic tremor. In addition, neither microinjection of D_1/5_ nor D_3_ antagonist into the striatum altered the induction of nicotine tremor. Thus, our results indicate that, unlike parkinsonian resting tremor, striatal dopamine receptors are not involved in regulation of nicotine-induced kinetic tremor.

In the D_2_ receptor family, D_3_ receptors are highly expressed in the cerebellum in addition to cerebral cortex, limbic regions, and striatum (Sokoloff et al., 1990; Bouthenet et al., 1991; Lévesque et al., 1992; Diaz et al., 2000; Kim et al., 2009). The cerebellum receives dopaminergic neurons from the red nucleus, substantia nigra, and ventral tegmental area (Kizer et al., 1976; Kosmowska and Wardas, 2021), and contains a high density of D_3_ receptors whereas the levels of D_1_, D_2_, D_4_, and D_5_ receptors are low (Diaz et al., 2000; Kosmowska and Wardas, 2021). We and other groups showed that cerebellar D_3_ receptors regulated spontaneous locomotor activity and the induction of extrapyramidal movement disorders (i.e., catalepsy) (Barik and de Beaurepaire, 1996, 2005; Kolasiewicz et al., 2008; Shimizu et al., 2014). Interestingly, the present study demonstrated that microinjection of the D_3_ antagonist U-99194 suppressed nicotine-induced tremor generation. Our results strongly suggest that cerebellar D_3_ receptors positively control the induction of nicotine tremor mediated by the olivo-cerebellar neural pathway.

We previously showed that nicotine-induced tremor was of IO origin and alleviated by medications for essential tremor (e.g., propranolol, diazepam, and phenobarbital) (Kunisawa et al., 2016; 2018), suggesting that nicotine-induced tremor is useful as a model of essential tremor. Essential tremor is one of the common neurological disorders affecting approximately 1% of people worldwide (Puschmann and Wszolek, 2011; Kosmowska and Wardas, 2021). Although several drugs are applicable in its treatment, there are no specific drugs for essential tremor, and approximately one third of patients are treatment-resistant. The present study suggests that the selective antagonists for D_3_ receptors or agonists for D_1/5_ receptors may have a therapeutic potential. Interestingly, linkage analysis of genes associated with essential tremor identified four loci: chromosome 3q13 (*ETM1*) (Gulcher et al., 1997), 2p22-p25 (*ETM2*) (Higgins et al., 1997), 6p23 (*ETM3*) (Shatunov et al., 2006), and 5q35 (Hicks et al., 2016), among which *ETM1* contains the *DRD3* gene encoding D_3_ receptors (Gulcher et al., 1997). In addition, *DRD3-Gly* (Ser9Gly variant), which shows a greater affinity for dopamine than the non-mutated form, is reportedly associated with the risk of essential tremor (Jeanneteau et al., 2006). Thus, dopamine D_3_ receptors may be involved in the modulation of essential tremor. Taken together with the present results, dopamine D_3_ receptor antagonists may be useful for the treatment of essential tremor. Further studies are required to validate this hypothesis and clarify the role of dopaminergic neurons in the pathogenesis of essential tremor.

## Data Availability

The raw data supporting the conclusion of this article will be made available by the authors, without undue reservation.
